# Molecular Dynamics Simulation of the Complex PBP-2x with Drug Cefuroxime to Explore the Drug Resistance Mechanism of *Streptococcus suis* R61

**DOI:** 10.1371/journal.pone.0035941

**Published:** 2012-04-26

**Authors:** Yan Ge, Jiayan Wu, Yingjie Xia, Ming Yang, Jingfa Xiao, Jun Yu

**Affiliations:** 1 CAS Key Laboratory of Genome Sciences and Information, Beijing Institute of Genomics, Chinese Academy of Sciences, Beijing, China; 2 College of Biological Sciences, China Agricultural University, Beijing, China; 3 State Key Laboratory of Genetic Resources and Revolution, Kunming Institute of Zoology, Chinese Academy of Sciences, Kunming, China; 4 Graduate University of Chinese Academy of Sciences, Beijing, China; Universität Erlangen-Nürnberg, Germany

## Abstract

Drug resistance of *Streptococcus suis* strains is a worldwide problem for both humans and pigs. Previous studies have noted that penicillin-binding protein (PBPs) mutation is one important cause of β-lactam antibiotic resistance. In this study, we used the molecular dynamics (MD) method to study the interaction differences between cefuroxime (CES) and PBP2x within two newly sequenced *Streptococcus suis*: drug-sensitive strain A7, and drug-resistant strain R61. The MM-PBSA results proved that the drug bound much more tightly to PBP2x in A7 (PBP2x-A7) than to PBP2x in R61 (PBP2x-R61). This is consistent with the evidently different resistances of the two strains to cefuroxime. Hydrogen bond analysis indicated that PBP2x-A7 preferred to bind to cefuroxime rather than to PBP2x-R61. Three stable hydrogen bonds were formed by the drug and PBP2x-A7, while only one unstable bond existed between the drug and PBP2x-R61. Further, we found that the Gln569, Tyr594, and Gly596 residues were the key mutant residues contributing directly to the different binding by pair wise energy decomposition comparison. By investigating the binding mode of the drug, we found that mutant residues Ala320, Gln553, and Thr595 indirectly affected the final phenomenon by topological conformation alteration. Above all, our results revealed some details about the specific interaction between the two PBP2x proteins and the drug cefuroxime. To some degree, this explained the drug resistance mechanism of *Streptococcus suis* and as a result could be helpful for further drug design or improvement.

## Introduction

The worldwide *Streptococcus suis* pathogen is a serious problem for both humans and pigs. This pathogen can lead to meningitis, endocarditis, septicemia, septic arthritis, pneumonia, and abortion in pig, and it can subsequently infect humans via direct contact [1]–[3]. To treat or resist this pathogen, multi-antimicrobials have been used extensively in recent years. Multi-antimicrobials, particularly β-lactam antibiotics, are the most popular antibiotics owing to their comparatively high efficiency, low cost, delivery convenience, and minimal side effects [4]. However, drug resistance often arises to these antimicrobials [5]–[11]. Further complicating the problem, the number of newly designed drugs to treat or resist this pathogen continually decreases [12]. Therefore, intensive study of the specific drug resistance mechanism of *Streptococcus suis* is of utmost importance for further research and treatment of the pathogen.

Previous studies have noted that target protein mutation is a decisive reason for drug resistance. Within the protein category, PBPs proteins, which play essential roles in constructing bacteria's cell walls, are particularly important [13]–[18]. In the β-lactam-resistant *S. pneumonia*, the coding genes of PBPs introduce many substitutions throughout the entire protein. This subsequently causes these genes to lose their affinity with the antibiotics [19]–[22]. Based on an MIC test, Hu *et al.*'s research data indicated that *Streptococcus suis* R61 was resistant to β-lactam. The reason for this resistance may be attributed to the mutation of the drug's target protein: PBP2x [23]. Here, we investigated the specific interaction between PBP2x and cefuroxime (CES) in two *Streptococcus suis*: the drug-sensitive strain A7 (*S. suis* A7), and the drug-resistant strain R61 (*S. suis* R61). We did so using the molecular dynamic method (MD).

MD simulation is a powerful tool for studying the binding mechanism at the atomic level [24]–[28]. In this study, we used MD simulation to investigate the detailed interactions between PBPs and CES in order to explore the drug resistance mechanism. First, we constructed the complex structure of PBP-2x proteins and cefuroxime by Modeller [29]. With the help of the Amber software package [30], we carried out a comparative dynamics study of the binding mode between two different proteins and their interaction with the drug. Our research returned several useful conclusions that could be conducive to understanding the drug resistance mechanism of *Streptococcus suis*. Further, these conclusions may be beneficial to further drug design for protection of infected animals and humans.

## Methods

### Model Construction

Four PBPs were identified by sequence similarities searching in *S. suis* R61 and *S. suis* A7: pbp2x, pbp2b, pbp1a, and pbp2a. A previous report demonstrated that PBP2x is the primary PBP target in β-lactam-resistant *S. pneumoniae* strains [13]. Further, PBP2x in the drug resistant R61 strain here showed more mutations than that in the *S.suis* A7 and that in the other *S. suis* [23]. In this study, we adapted the homology modeling method to construct the three-dimensional structures of the target proteins. A crystallized complex consisting of the PBP2x protein and the cefuroxime in *Streptococcus pneumoniae* (PDB code 1QMF) has been reported in a previous study [13]. Recently, this complex structure has allowed research involving resistance pattern analysis of *S.suis* R61 to β-lactam antibiotics to take place [23]. Accordingly, we made use of this complex structure (PDB code 1QMF) to produce our target protein. Unfortunately, the N terminal region from 1 to 200 has not been clearly and continuously resolved. As a consequence, the N terminal region was not completely included in our modeled structures. This is an unavoidable shortcoming within our research structure due to an external constraint. However, the two proteins did have complete transpeptidase and C terminal regions, in which the mutant sites of the drug-sensitive strain R61 compared to the drug-resistant strain A7 mostly located. The former region was proven to be the functional area for drug interaction. Further, the MD study indicated that the whole system reached equilibrium after about 8 ns. This could be verified by the RMSD plot of both the complex and the drug molecule. It could also be verified by other indices like Temperature, Density, and Total Energy (Data not shown). This meant that our further analysis was reasonable. This further indicated that the whole study would be helpful for exploring the binding characters between the PBP2x proteins and the cefuroxime (CES) in the drug-resistant and drug-sensitive strains.

The sequence alignment of the target protein and the template protein was performed using Modeller V8.1 and the Blosum 62 Matrix. We adopted the basic modeling method to produce the structures, leaving the loop regions optimized in the further minimization and MD stage. The pdb files of model proteins were generated for each target protein using spatial structure restraints based on the sequence alignment data. Then the Procheck [31], Dope [32], and Ga341 [33] methods were applied to evaluate the quality of these candidate models. For each modeled structure produced, the Procheck, Dope, and Ga341 methods gave an evaluation score. Procheck identified the exact percentage of amino acids located in the allowed regions. Dope and Ga341 scores provided an evaluation index integrated in the evaluation modules of Modeller to assess the produced model's quality. This could be achieved by the log file in running Modeller. The higher these scores were, the better the quality of the structure was assessed to be. We chose the model that had the highest score under these evaluation criteria, and then we performed the refinement step. First, 5000 iterations of the steepest descent (SD) calculation were carried out. Then, the conjugated gradient (CG) calculation was implemented until the convergence on the gradient reached 0.05 kcal/(Å mol). After simulations were performed, the homology models were obtained via the molecular dynamics (MD) calculation. This was done in the Amber software package under the AMBERff99SB force field to proceed with the optimization. The rectangular box used in Amber by Xleap to add water molecules to solve the simulated complex is not cubic and it is infinitely repeated in space by the periodic boundary method, leading the ends of the complex close to their periodic images. If a list of numbers are specified to the solvateBox command in Xleap to make the box cubic, the simulated complex will be significantly added more water; consequently, the calculation will slow down enormously. In terms of reducing the problem of solute rotation, a more efficient shaped box of water to use would be a truncated octahedron [34]. Hence, we add a truncated octahedral box of water to solve the protein and keep the simulation temperature at 300 K. Finally, a conjugate gradient energy minimization of the full protein was performed until the root mean-square (rms) gradient energy was lower than 0.001 kcal/(Å mol). The quality of the initial protein model was improved after completion of all the above steps.

The complex structure involving PBP2x and cefuroxime was produced by sequence alignment data on the basis of the template protein (PDB code 1QMF). The geometry of the drug was optimized at the HF/6-31G level. Partial atomic charges of the cefuroxime molecule were assigned by DFT calculations at the B3LYP/cc-pVTZ level and fitted with the RESP procedure [35]. The atom types were assigned using the Antechamber module [36] in Amber 9. We chose the Antechamber module to obtain the topology file of the drug for the subsequent Amber simulation. At this point the entire structure of this complex was ready for MD simulation.

### Molecular Dynamic (MD) Simulation

MD simulations were performed at the molecular mechanics level using the Amber 9 program package with the AMBERff99SB force field [30]. Structures of the complex models were solvated in a truncated octahedral box of TIP3P water that extended at least 12 Å in each direction in the solute. To calculate the nonbonded interactions, the cut-off distance was kept at 12 Å. Before MD simulations took place, the systems were relaxed by a series of steepest descent (SD) and conjugated gradient (CG) minimizations. MD simulations were applied to these minimized systems by gradually heating over 50 ps from 0 to 300 K with the protein backbone atoms fixed using a force constant of 2 kcal/(mol Å^2^). Then, we carried out 50 ps density equilibration with the same restraints on the protein backbone. The following step was constant pressure equilibration at 300 K. The periodic boundary was used to keep the pressure constant by setting the corresponding parameters: PRES0 = 1.0, NTP = 1, and TAUP = 2.0. Isotropic position scaling was used to maintain the pressure (NTP = 1); a constant pressure periodic boundary with an average pressure of 1 atm (PRES0) and a relaxation time of 2 ps were used (TAUP = 2.0).

The two complexes in the study were very large, especially when the water molecules were added to solvate the complex. For reasons of computational time and resource use, we simulated each of the two complexes for 16 ns to explore the binding difference. Also, after simulation for 16 ns, the trend became clear for both complexes. That is, the RMSD value indicated that the complexes began to converge from approximately 8 ns. Other indices such as Temperature, Density, and Total Energy all proved that the system converged in the whole equilibrium period (Data not shown). All simulations were performed under periodic boundary conditions, and long-range electrostatics were treated using the Particle-Mesh-Ewald method. The time step was set to 2 fs and the trajectory was recorded every 2 ps with shake on hydrogen atoms. For temperature control we used the Langevin thermostat (NTT = 3) to maintain the temperature of our system at 300 K. This temperature control method used Langevin dynamics with a collision frequency given by GAMMA_LN = 1.0.

### MD Analysis

To assess the stability of MD trajectories, the root-mean-square deviation (RMSD) of the complex backbone atoms relative to the starting structure during the MD production phase was calculated. The calculation was performed under the PTRAJ module in the Amber program suite. MD simulations can explicitly analyze hydrogen bond properties, such as donor-acceptor assignments and hydrogen bond occupancies. The criteria to identify hydrogen bonds include: (1) the distance between proton donor (X) and acceptor (Y) atoms is less than or equal to 3.5 Å; and (2) the angle X-H…Y is greater than or equal to 120°. Here, special attention was given to stable hydrogen bonds, which hold this mode for at least 40% of the simulation period [37].

The binding free energy between the protein and the drug was computed using the MM-GBSA (Molecular Mechanics Generalized Born Surface Area) and MM-PBSA (Molecular Mechanics Poisson–Boltzmann Surface Area) methods in Amber SANDER modules [38]. Early versions of Amber require an external poisson Boltzmann solver such as Delphi, but it was possible to use the internal PBSA program in the software used in this study. The GB method was determined by the IGB parameter. Here we set gb = 2 by turning on the Onufriev, Bashford, Case (OBC) variant of GB with a = 0.8, b = 0.0, and g = 2.909 [39]. Nonpolar contribution to the solvation free energy, represented by PBSUR/GBSUR in Table 1, was calculated using solvent-accessible-surface-area-dependent terms [40]–[41]. The surface area is computed with Paul Beroza's molsurf program, which is based on analytical ideas primarily developed by Mike Connolly [42].

**Table 1:** Binding free energies (kcal/mol) between PBP2x protein and cefuroxime (CES) in the two complexes.

Method	Contribution	Complex	
		PBP2x-R61-CES	PBP2x-A7-CES
MM	ELE	−12.7±3.9	−24.0±4.9
	VDW	−41.6±3.3	−58.1±2.9
	INT	0.0±0.2	0.0±1.1
	GAS	−54.2±4.9	−82.1±5.9
PBSA	PBSUR	−5.9±0.3	−6.1±0.1
	PBCAL	42.9±5.9	51.7±4.3
	PBSOL	37.0±5.8	45.6±4.3
	PBELE	30.2±4.1	27.7±2.9
	PBTOT	−17.2±4.2	−36.4±4.9
GBSA	GBSUR	−5.9±0.3	−6.1±0.1
	GB	31.7±3.5	39.6±3.6
	GBSOL	25.8±3.4	33.5±3.6
	GBELE	19.0±2.0	15.6±2.3
	GBTOT	−28.4±3.1	−48.6±3.7

ELE, electrostatic interactions; VDW, van der Waals interactions between the fragments; INT, internal energy arising from bond, angle and dihedral terms in the MM force field; GAS, total gas phase energy (sum of ELE, VDW and INT); PBSUR/GBSUR, nonpolar contribution to solvation; PBCAL/GB, the electrostatic contribution to the solvation free energy calculated by PB or GB respectively; PBSOL/GBSOL, sum of nonpolar and polar contributions to solvation; PBELE/GBELE, sum of the electrostatic solvation free energy and MM electrostatic energy; PBTOT/GBTOT, estimated total binding free energy calculated from the terms above.

In this approach, the trajectory frames were stripped off of counter-ions and water molecules. Further, the decomposed interaction energy of each residue was calculated to obtain a detailed view of the specific binding. These decompositions are possible for molecular mechanics and free energy of solvation, but not for entropies. The details of the underlying theory are described elsewhere [43].

## Results and Discussion

### Sequence Alignments and Model Construction of the Complex

**Figure 1:**
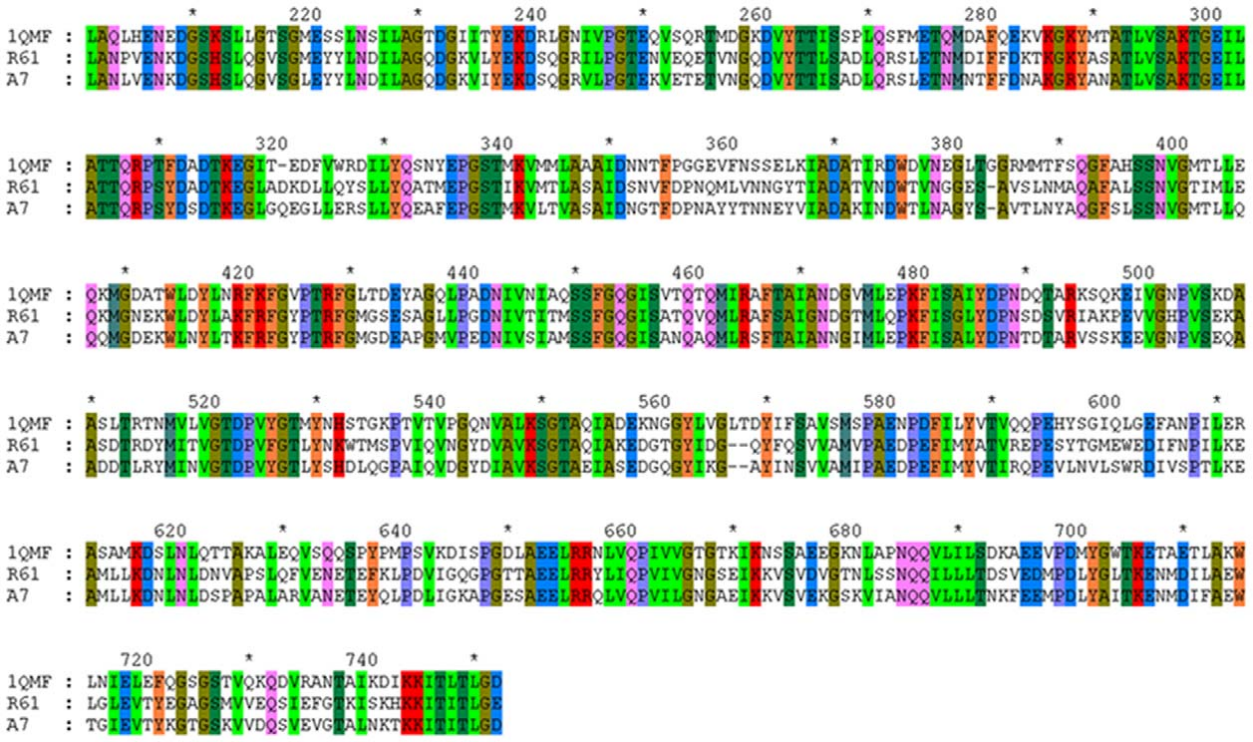
Sequence alignment between PBP2x-R61 (R61), PBP2x-A7 (A7), and the template protein (PDB code 1QMF).

**Figure 2:**
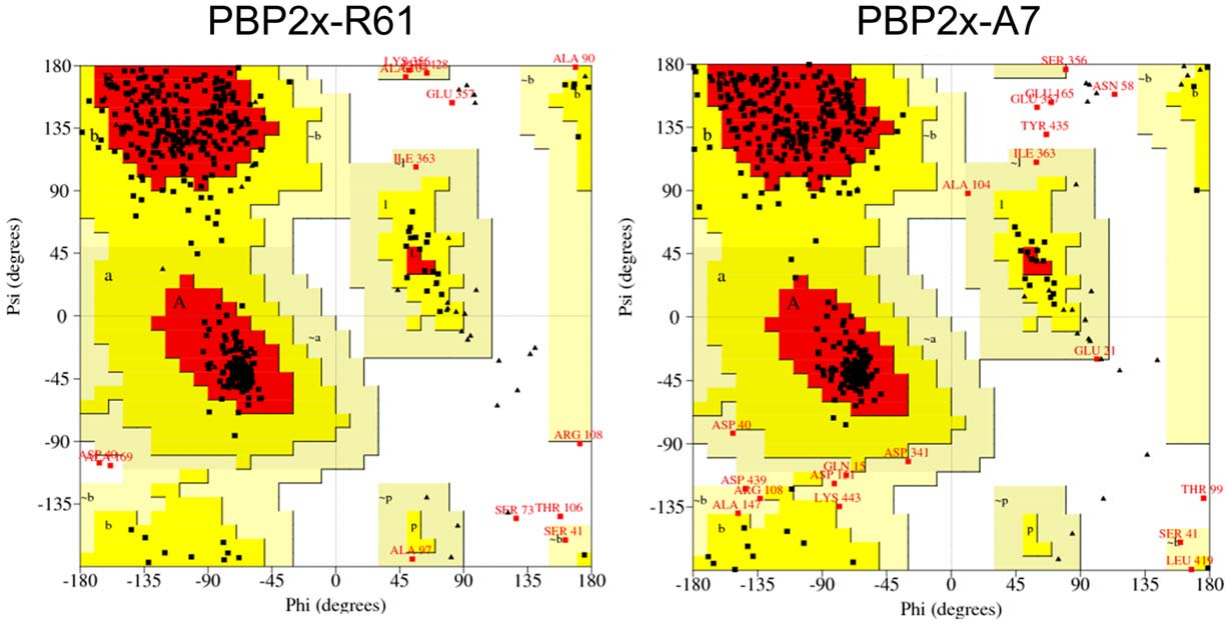
Ramachandran plot of modeled PBP2x-R61 and PBP2x-A7. The most favored regions are red. Additionally allowed, generously allowed, and disallowed regions are indicated as yellow, light yellow, and white, respectively.

The sequence alignment of PBP2x-R61, PBP2x-A7, and the template protein (PDB code 1QMF) were obtained by ClustalW [44] and viewed using Genedoc [45]–[47] (Figure 1). It could be seen that PBP2x-R61 and PBP2x-A7 were both very similar to the template. The identities of PBP2x-R61 and PBP2x-A7 compared to the template were 56.70% and 56.34%, respectively. Amongst homologues, protein structures are more conserved than protein sequences, but sequences falling below a 25% sequence identity could have a very different structure [48]. Additionally, evolutionarily-related proteins have similar sequences, and naturally occurring homologous proteins have similar protein structures. However, due to sequence conservation, the three-dimensional protein structure tends to be evolutionarily more conserved than it is expected to be [49]. Further, in Modeller, a sequence identity value above approximately 25% generally indicates a potential template. This means that it is capable of producing the target structures with the homology modeling method. The qualities of the model proteins here were evaluated by Procheck (Figure 2). The Ramachandran plot showed that 84.5% PBP2x-R61 residues and 85.0% PBP2x-A7 residues were located in the most favored regions. 12.8% PBP2x-R61 and 11.0% PBP2x-R61 residues were located in additionally allowed regions. This outcome was comparable with the high-quality crystallographic structures determined at a resolution of 2.8 Å. Considering further optimization would be executed by Amber, this result suggested that the backbone conformations of these two complexes were acceptable.

**Figure 3:**
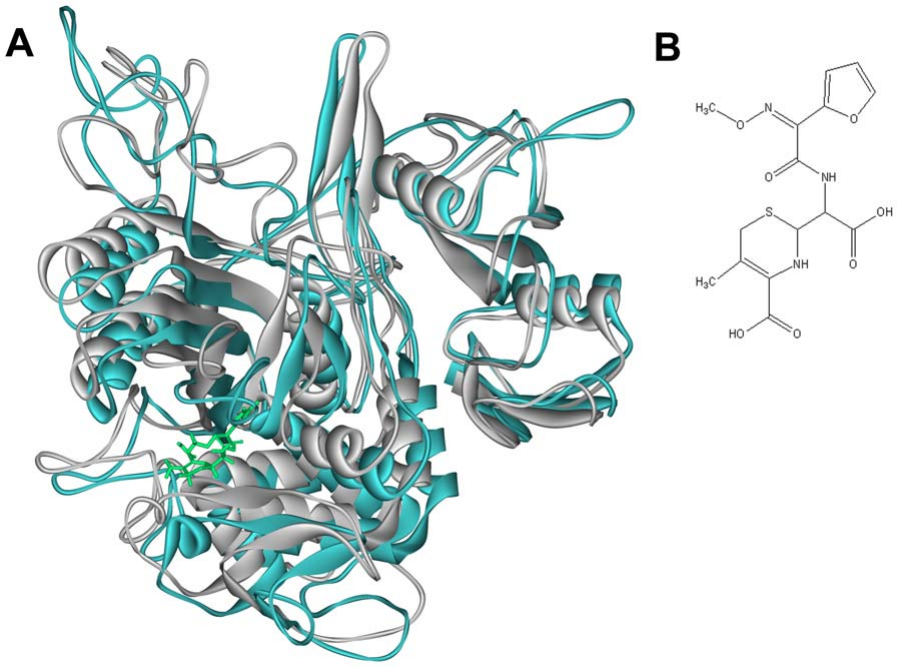
Comparison of PBP2x-R61 and PBP2x-A7. (A) The superimposition of initial structures of the complex between PBP2x-R61 (white color) and PBP2x-A7 (cyan color) with the drug (green color). (B) The structure of the cefuroxime drug.

The produced three-dimensional structures of the two proteins had a central transpeptidase domain (residues 265–619), which is the classical class A structure of the PBP protein [50]. It has been reported that the central domain is the active site area and plays an important role in drug interaction [13]–[14], [51]. We could see that this central domain region surrounded the drug molecule (Figure 3A). Although the figure showed that the two-modeled proteins were very similar to one another, the RMSD (root-mean-squared-deviation) value was 5.57 Å. This indicated the original influence of the mutated residues. In fact, there were 165 total mutated sites of PBP2x-R61 compared to PBP2x-A7. Most of them were located in the loop regions of the transpeptidase and C-terminal domain.

### Stability of the Complex's MD Trajectories

**Figure 4:**
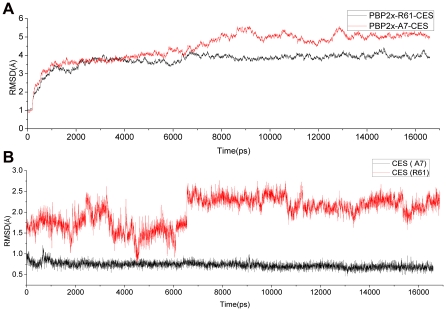
RMSD values for PBP2x-R61-CES and PBP2x-A7-CES relative to the starting structure during MD simulation. (A) The whole backbone atoms. (B) The drug molecule.

In this study, we simulated more than 16 ns to ensure that the equilibration phase lasted long enough for further binding energy and hydrogen binding mode analysis. The stability was evaluated by the RMSD values of the backbone atoms in the two complexes (Figure 4A) and those of the drug molecule alone (Figure 4B) relative to those in the starting structure of the heating phase. The trajectories of the whole complex were stable after simulation for about 8 ns (Figure 4A). Snapshots from this time through the end were extracted for further hydrogen bond interaction and binding energy analysis. In total, more than 4000 snapshots were obtained, and this amount was adequate for assessment of the whole binding property of the two complexes.

It could easily be seen that the drug in the drug-sensitive R61 strain was much more flexible than the drug in the drug-resistant A7 strain (Figure 4B). This may indicate that the mutations had a large effect on the interaction with the protein. However, after nearly 7 ns, the drug in the R61 strain began to converge with the RMSD value, which fluctuated around 2.25 Å. This probably suggests that the snapshots we extracted for computation were reasonable because the whole complex and drug were equilibrated.

### Binding Energies

We simulated the two complexes all for as long as 16 ns and recorded the coordinate every 2 ps. As the equilibrium time was 8 ns, there were a total of 4,000 snapshots. For energy computation, we chose the snapshot with an interval of 50, and got a total of 80 representative snapshots. Due to the long intervals between each snapshot, they were considered independent and non-correlate. Thus, due to their ability to represent the whole equilibrium process, they could be utilized to calculate the overall binding energy.

MM-PBSA analysis was performed to evaluate the general binding activity. Since these model structures were homology modeling structures, the absolute values of these energies could vary. The interaction energies corresponded only to the enthalpy contributions of binding free energy. This could be found by performing normal mode analysis. But, as we were studying two proteins binding to the same ligand, a comparison of states of similar entropy was desired; we did not take the entropy contributions into consideration in practice. Another reason for this was that normal mode analysis calculations were computationally expensive and tended toward a large margin of error. This would thus introduce significant uncertainty into the result. However, even with these qualifications, the relative importance of each protein or residue could be inferred by its rank order of interaction energy [52]–[53].

The binding energy results with standard deviations for every energy item are shown in Table 1. Obviously, the fluctuation for each item was reasonable. From the PBTOT or GBTOT item, it could be easily seen that the difference between the overall binding energy for the two complexes was very significant (36.4−17.2 = 19.2 kcal/mol). Even when taking the standard deviation into consideration, the difference was still very distinct; this confirmed that our comparison result was convincing. The absolute energy contrast clearly indicated the huge binding affinity difference; that is, the drug bound more tightly to PBP2x-A7 than to PBP2x-R61. As a result, cefuroxime could prevent this protein from executing its function in constructing the cell wall; subsequently, it could kill the bacteria. But in *S. suis* R61, this combining interaction was not strong enough to keep the protein bound to the drug and keep the strain from functioning. The main difference of the total energies could be attributed to the preferable binding van der Waals and electrostatic terms (Table 1). The total difference of these two parts reached 27.9 kcal/mol.

### Energy Decomposition of Each Amino Acid Residue

**Figure 5:**
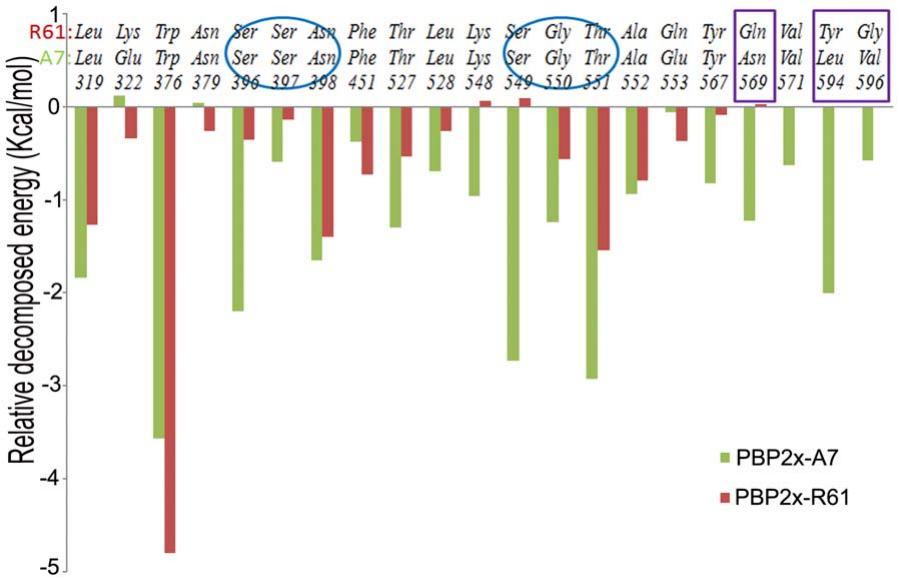
Relative decomposed energies of the corresponding residues between PBP2x-R61-CES and PBP2x-A7-CES. The ovals characterize the two active site areas and the blocks mark the three essential mutated residues.

The individual energy decompositions of all residues in the two complexes were calculated in order to qualitatively find the key residue that played a more important role in the cefuroxime binding. Although the decomposition values were not so quantitatively accurate, they did give a good indication for the comparison of the corresponding residues. This was helpful for location of the critical amino acids. Table 2 lists the residues for which the absolute energy was larger than 1.0 kcal/mol or for which the difference between the two complexes was larger than 0.5 kcal/mol. The absolute binding energy values of the conserved Leu319, Trp376, Asn398, and Thr551 were very large in the two complexes and this may reveal the universality of PBP protein interaction with the drug. In contrast, the conserved Ser396, Thr527, Ser549, and Gly550 had significantly different actions in the two complexes and these residues preferred to bind more to the drug for PBP2x-A7 than to the drug for PBP2x-R61. We propose that this explicit difference owes to the general conformation change of the two complexes. In fact, the RMSD values of these two proteins increased to 7.26 Å, even before the complete rotation of the drug molecule was considered (Data not shown). By carefully investigating the mutated sites, the Asn569, Leu594, and Val596 residues in PBP2x-A7 had much more binding contribution than did the corresponding Gln569, Tyr594, and Gly596 residues in PBP2x-R61. Besides these favoring PBP2x-A7 residues, the Glu322 and Glu553 residues bound to the drug more weakly than did the corresponding mutated PBP2x-R61 residues, Lys322, and Gln553. However, this kind of residue was seldom, and the whole binding energy profile proved that PBP2x-A7 generally bound much more tightly to cefuroxime (Figure 5). In a word, these mutated residues played an important role in changing the binding mode between *S. suis* R61 and the drug by notable different binding energy contributions.

**Table 2:** The list of the residues with absolute energy larger than 1.0 kcal/mol or the residues with the difference between the two complexes larger than 0.5 kcal/mol.

Position	PBP2x-A7-CES		PBP2x-R61-CES	
No.	Residue	Energy(kcal/mol)	Residue	Energy(kcal/mol)
319	Leu	−1.84	Leu	−1.27
322	Glu	0.17	Lys	−0.34
376	Trp	−3.57	Trp	−4.80
396	Ser	−2.20	Ser	−0.35
398	Asn	−1.65	Asn	−1.40
527	Thr	−1.30	Thr	−0.54
548	Lys	−0.96	Lys	0.06
549	Ser	−2.73	Ser	0.09
550	Gly	−1.24	Gly	−0.56
551	Thr	−2.93	Thr	−1.55
567	Tyr	−0.82	Tyr	−0.09
569	Asn	−1.23	Gln	0.02
571	Val	−0.63	Val	−0.01
594	Leu	−2.01	Tyr	−0.01
596	Val	−0.58	Gly	0.01

Furthermore, we paid attention to the residues 396–398 and 549–551 (Figure 5). These two regions were reported as the conserved amino acid motifs acting as the active site of this enzyme [13], [54]. These two regions contributed much more in binding to the drug in *S. suis* A7, while in *S. suis* R61 these residues had a much lower binding ability (Table 2). In consequence, the final drug resistance in *S. suis* R61 may due to the active site of PBP protein, which is not so active in the R61 strain as in the sensitive strain.

### Hydrogen Bond Interaction

**Figure 6:**
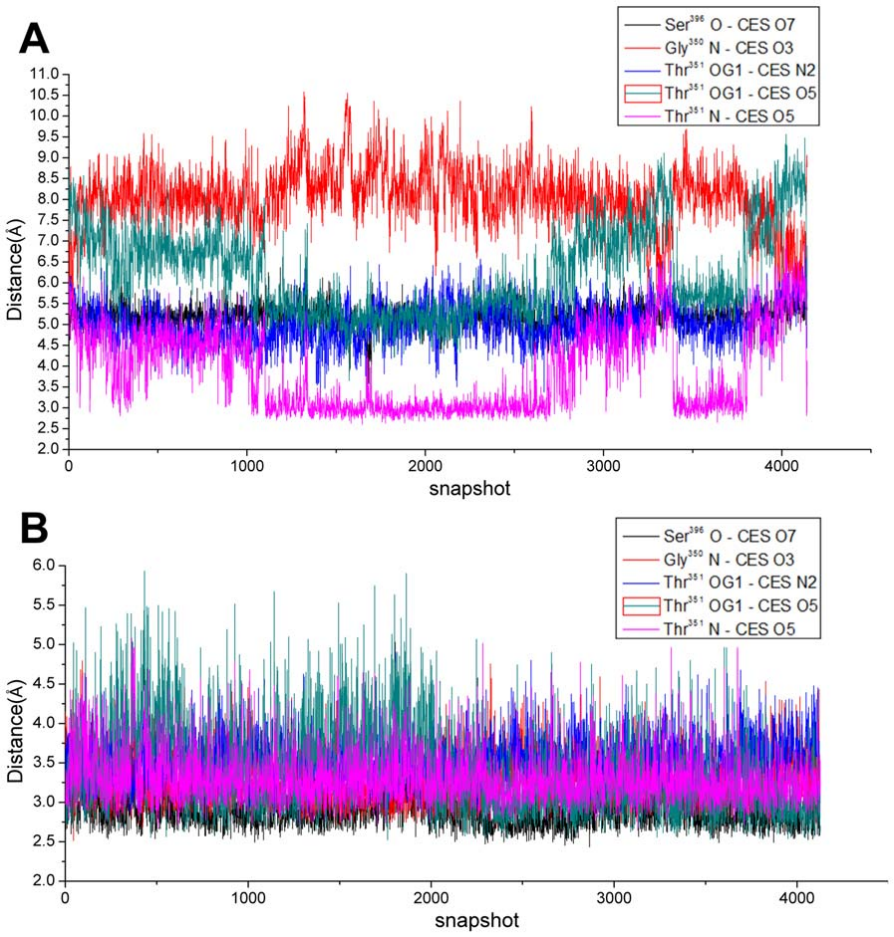
The distance variation of the five hydrogen bonds during MD simulation. (A) for PBP2x-R61-CES. (B) for PBP2x-A7-CES. Bond 1: Ser396 O - CES O7; Bond 2: Gly350 N - CES O3; Bond 3: Thr351 OG1 - CES N2; Bond 4:Thr351 OG1 - CES O5; and Bond 5: Thr351 N - CES O5.

There were four hydrogen bonds in total between the protein and the drug in the complex PBP2x-A7-CES, while there was only one hydrogen bond in the complex PBP2x-R61-CES. Three residues of Ser396, Gly550, and Thr551 formed those four hydrogen bonds in PBP2x-A7-CES, and the last residue contributed two hydrogen bonds. By plotting the distance of all five hydrogen bonds in these two systems in the whole equilibration process, it could be seen that bonds 1–4 were very stable with cefuroxime in the complex PBP2x-R61 during the whole simulation process (Figure 6A). But, the distance of hydrogen bond Thr351 N-CES O5 was fluctuant and the angle could not satisfy the criteria. Figure 6B shows the distance variation results of these five hydrogen bonds in the PBP2x-A7-CES complex. For bonds 1–4, the PBP2x-R61-CES distance was much longer than the corresponding distances in the complex PBP2x-A7-CES. This hydrogen bond distinction further verified the more efficient binding to the drug for PBP2x-A7. With this analysis, we could thus conclude that the hydrogen bond differences of the active site residues greatly contributed to the final drug resistance change for *S. suis* R61.

Although the Ser396, Gly550, and Thr551 residues were conserved in the two proteins, they formed totally different hydrogen binding networks in the two complexes. We speculate this difference in hydrogen bond interaction may be attributed to the structure conformation alteration. This change was also verified by the energy contribution (Table 2, Figure 5). The decomposed energies of these three residues in the PBP2x-A7-CES complex were −2.20 kcal/mol (Ser396), −1.24 kcal/mol (Gly550), and −2.93 kcal/mol (Thr551). The respective values in the complex PBP2x-R61 with cefuroxime were −0.35 kcal/mol, −0.56 kcal/mol, and −1.55 kcal/mol. These residues may be a key factor in drug resistance change for *S. suis* R61 to cefuroxime.

### Hydrophobic Contact

**Figure 7:**
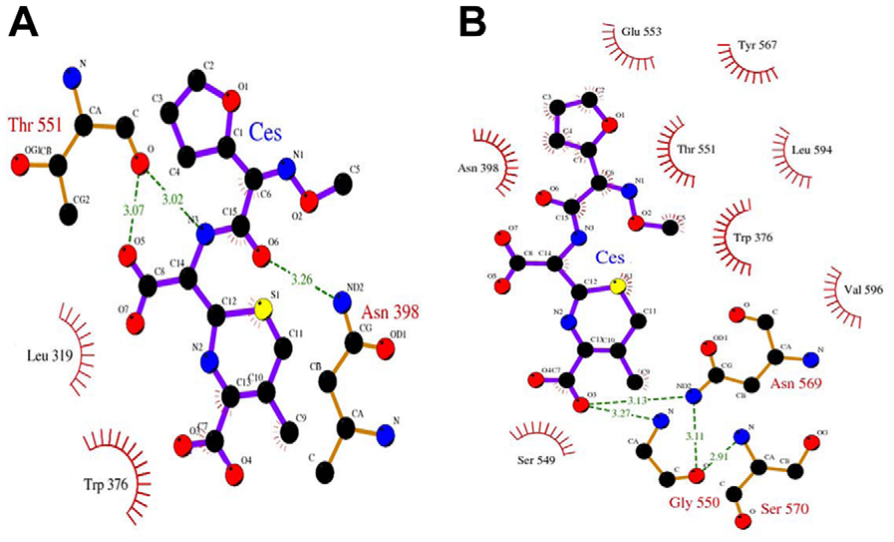
LIGPLOT representation of cefuroxime (CES) binding to PBP2x-R61 and PBP2x-A7. Ligand, protein, and hydrogen bonds are in thick blue, thick brown, and broken green lines, respectively. Residues involved in hydrophobic contact are associated with a curved comb. (A) Open form of cefuroxime (CES) bound to PBP2x-R61. (B) Open form of cefuroxime (CES) bound to PBP2x-A7.

The VDW item of the MM-PBSA and MM-GBSA in Table 1 mostly represents the nonpolar interaction: the hydrophobic contact between the molecules. Though the two complexes, PBP2x-A7-CES and PBP2x-R61-CES, all have large hydrophobic effects, the former has an obviously much high contribution. This can also be found by the decomposed energy contribution (Table 2 and Figure 5). Residues Leu319, Trp376, Phe451, Leu528, Ala552, and Val571 in the two complexes all played a great role in the hydrophobic contribution, while the residues in PBP2x-A7-CES generally had a high value. Noticeably, mutant sites of Tyr594 and Gly596 in PBP2x-R61-CES had a much lower value compared to the corresponding hydrophobic amino acids Leu594 and Val596 in PBP2x-A7-CES. A similar trend is also shown in the figure illustration in Figure 7. Overall, the hydrophobic interaction was prevalent in the two studied complexes, but it was more prominent for PBP2x-A7-CES. This probably indicates a much higher binding affinity for PBP2x-A7 combining with the drug than for PBP2x-R61. This result was in accordance with the final resistance phenomenon.

### Binding Mode Variation

The residues with the higher decomposed energy were found all around the drug molecule; thus we investigated the residues apart from the drug within 7 Å in the two systems. By calculating the distance, we found that for the complex PBP2x-R61-CES in the initial state before MD simulation, there were 40 residues. There were 34 residues in the PBP2x-A7-CES complex. After MD simulation, the averaged structure for each system during the whole equilibration phase was extracted to illustrate the binding property. At this time, there were 27 residues apart from the drug within 7 Å in the PBP2x-R61-CES complex VS 37 residues in the PBP2x-A7-CES complex. In the 27 residues of PBP2x-R61-CES, 23 residues formed 47 hydrogen bonds in the protein itself, while 36 residues in PBP2x-A7-CES formed 81 hydrogen bonds. This demonstrated that the intermolecular interaction of the residues around the drug was more popular in PBP2x-A7-CES than in PBP2x-R61-CES. This probably changes the overall structure conformation of this area and subsequently affects the interaction between the PBP2x and the drug. These results indicated that PBP2x-R61 had a looser interaction with the drug molecule, and the binding mode exhibited combination differences with cefuroxime as compared to PBP2x-A7 (Figure 7). Figure 7 gives an explicit exhibition of the interaction profile between the residues and the drug, according to the averaged structure. There were more residues in PBP2x-A7 than in PBP2x-R61, and these residues compactly surrounded the drug and interacted with it. In addition, we listed the mutated residue pairs between PBP2x-R61 and PBP2x-A7 in this region (Table 3). Compared with the residues in PBP2x-A7, the mutated residues 320, 321, 322, 553, and 596 in PBP2x-R61 changed their property. The residues 373, 568, 569, and 595 remained the same. We speculate that these residues were responsible for the different drug-resistance phenomenon because they changed the topology conformation. The Gln569 and Gly596 residues, however, contributed largely by direct binding. The variation of the binding mode induced by mutation was in agreement with the previous conclusions drawn from hydrogen bond and energy analysis.

**Table 3:** The property of the mutated residues in the binding site of the two complexes PBP2x-R61-CES and PBP2x-A7-CES.

Position	Residue		Property		Energy (Kcal/mol)		H-Bonds –Number	
No.	A7	R61	A7	R61	A7	R61	A7	R61
320	Gly	Ala	Polar	Non-polar	0.13	0.02	2	1
321	Gln	Asp	Polar	Acidic	0.00	−0.06	0	4
322	Glu	Lys	Acidic	Basic	0.17	−0.34	3	3
373	Ile	Val	Non-polar	Non-polar	−0.06	−0.02	2	2
553	Glu	Gln	Acidic	Polar	−0.06	−0.37	2	0
568	Ile	Phe	Non-polar	Non-polar	−0.15	−0.17	4	4
569	Asn	Gln	Polar	Polar	−1.23	0.02	4	0
595	Asn	Thr	Polar	Polar	−0.04	0.01	3	0
596	Val	Gly	Non-polar	Polar	−0.58	0.01	1	0

(A7: PBP2x-A7-CES; R61: PBP2x-R61-CES).

### Conclusion

Hu *et al.*'s research indicated that *S. suis* R61 was resistant to the drug β-lactam based on MIC test results. Further, the results indicated that the resistance may be caused by the mutation of the drug target PBP2x [23]. In order to study the drug-resistance mechanism, 16 ns MD simulation for the two complexes of PBP2x-A7 and PBP2x-R61 with cefuroxime were performed. By applying the molecular modeling method, we obtained some useful results about the detailed interaction between cefuroxime and the protein. The MM-PBSA result firmly proved the tighter binding to PBP2x-A7 as compared to the binding to PBP2x-R61. This indicated the reason for *S. suis* R61 drug resistance against cefuroxime. There were 4 stable hydrogen bonds supporting the tight interaction between PBP2x-A7 and the drug, while there was only one unstable hydrogen bond between PBP2x-R61 and the drug. Besides, the residues forming hydrogen bonds all belonged to the conserved active site area. This region has been reported to play an essential role in the interaction with the drug, and this can be further verified by energy decomposition analysis. As a result, the much lower contribution of the active site area in PBP2x-R61 was one important reason for *S. suis*'s drug resistance. Moreover, mutated sites 569, 594, and 596 were recognized as the key mutations. In PBP2x-A7 these residues contributed much more to drug binding to the drug, whereas they showed little contribution in PBP2x-R61. Because the structure conformation of the residues surrounding the drug in the binding site had a big difference, it could be concluded that the mutated residues induced the loose interaction between PBP2x-R61 and the drug. To our knowledge, our study has been the first investigation using the MD method to compare the specific interaction between PBP2x proteins and the drug in drug-sensitive and drug-resistant *Streptococcus suis*. These results will be helpful for further work on antibiotics and drug design.
